# Structural and immunological characterization of *E. coli* derived recombinant CRM_197_ protein used as carrier in conjugate vaccines

**DOI:** 10.1042/BSR20180238

**Published:** 2018-09-25

**Authors:** Ravi P.N. Mishra, Ravi S.P. Yadav, Christopher Jones, Salvatore Nocadello, George Minasov, Ludmilla A. Shuvalova, Wayne F. Anderson, Akshay Goel

**Affiliations:** 1 Biological E. Limited, Genome Valley, MN Park, Shameerpet, Hyderabad 500078, India; 2 National Institute for Biological Standards and Control (NIBSC), Blanche Lane, South Mimms, Potters Bar, Hertfordshire EN6 3QG, U.K.; 3 Center for Structural Genomics of Infectious Diseases, Northwestern University, Feinberg School of Medicine, Morton Bld. # 7-614 303 E. Chicago Ave., Chicago, IL 60611, U.S.A.

**Keywords:** CRM197, Carrier Protein, Conjugate Vaccine, X-ray crystallography, E. coli

## Abstract

It is established that the immunogenicity of polysaccharides is enhanced by coupling them to carrier proteins. Cross reacting material (CRM_197_), a nontoxic variant of diphtheria toxin (DT) is widely used carrier protein for polysaccharide conjugate vaccines. Conventionally, CRM_197_ is isolated by fermentation of *Corynebacterium diphtheriae* C7 (β_197_) cultures, which often suffers from low yield. Recently, several recombinant approaches have been reported with robust processes and higher yields, which will improve the affordability of CRM_197_-based vaccines. Vaccine manufacturers require detailed analytical information to ensure that the CRM_197_ meets quality standards and regulatory requirements. In the present manuscript we have described detailed structural characteristics of *Escherichia coli* based recombinant CRM_197_ (rCRM_197_) carrier protein. The crystal structure of the *E. coli* based rCRM_197_ was found to be identical with the reported crystal structure of the C7 CRM_197_ produced in *C. diphtheriae* C7 strain (Protein Data Bank (PDB) ID: 4EA0)_._ The crystal structure of rCRM_197_ was determined at 2.3 Å resolution and structure was submitted to the PDB with accession number ID 5I82. This is the first report of a crystal structure of *E. coli* derived recombinant CRM_197_ carrier protein. Furthermore, the rCRM_197_ was conjugated to Vi polysaccharide to generate Typhoid conjugate vaccine (Vi-rCRM_197_) and its immunogenicity was evaluated in Balb/C Mice. The Vi-rCRM_197_ conjugate vaccine was found to generate strong primary α-Vi antibody response and also showed a booster response after subsequent vaccination in mice. Overall data suggest that *E. coli* based recombinant CRM_197_ exhibits structural and immunological similarity with the C7 CRM_197_ and can be used as a carrier protein in conjugate vaccine development.

## Introduction

Over the past three decades, many routine childhood and adult vaccines have been developed using conjugation technology. Conjugate vaccines are developed by covalent attachment of an antigenic polysaccharide to a nontoxic carrier protein. Conjugation to a nontoxic carrier protein enhances the immunogenicity of polysaccharide antigens, enabling host defence against diseases caused by encapsulated pathogens. Conjugation transforms the T cell-independent response of polysaccharide vaccines to a T cell-dependent one. Conjugate vaccines have been demonstrated to be immunogenic and capable of inducing immunological memory and high avidity antibodies. Unlike unconjugated polysaccharides, conjugate vaccines also elicit protective responses in the immature immune system of young infants and the senescent immune system of the elderly [1–3].

Cross reacting material (CRM_197_) is widely used as a carrier protein in conjugate vaccines [3]. CRM_197_ protein is a nontoxic variant of diphtheria toxin (DT, molecular weight: ~58 kDa) with a single mutation (Glycine to Glutamate substitution at position 52) that eliminates its toxicity [4,5]. The protein nonetheless retains the same immunostimulant properties as DT and it is used to generate safe and effective polysaccharides conjugate vaccines for all age groups [6,7].

CRM_197_-based polysaccharides conjugate vaccines have already been developed to confer protection against important bacterial pathogens such as *Streptococcus pneumoniae* (Prevnar), *Haemophilus influenzae* type b (HibTITER, Vaxem-Hib) and *Neisseria meningitidis* serogroup A, C, Y and W-135 (e.g. Menveo and Menjugate). These vaccines have been successfully used to immunized hundreds of millions of people worldwide. Several conjugate polysaccharides vaccines using CRM_197_ as carrier protein are under preclinical and clinical evaluation. These include vaccines against *Staphylococcus aureus, Streptococcus agalactiae* (Group B *Streptococcus*), *Salmonella* Typhi, *Salmonella* Paratyphi, *Mycobacterium tuberculosis* and several pneumococcal conjugate vaccines [3,8–14].

CRM_197_ is synthesized as a single chain holoprotein, which comprises two domains fragment A (catalytic domain) and fragment B (transmembrane domain), bound together by a disulfide bridge. The B domain contains a subdomain for binding to the HB-EGF cell receptor and another subdomain for translocation into eukaryotic cells. *In vitro*, mild trypsin treatment and reduction divides the toxin into two functional moieties (the ‘nicked form’). The receptor-binding subdomain of fragment B has an all-β-sheet structure, in contrast with the transmembrane subdomain containing nine α-helices. Two disulfide bridges are present in the intact holoprotein: one bridge joins Cys^186^ to Cys^201^, linking fragment A to fragment B while a second bridge joins Cys^461^ to Cys^471^ within fragment B [15].

Since CRM_197_ is genetically nontoxic, chemical toxoiding is not required. This helps to preserve epitopes which would otherwise be lost. It has been suggested that preservation of T-helper epitopes explains the improved carrier effect of CRM_197_ compared with diphtheria toxoid [13]. The lack of toxicity in CRM_197_ has been examined *in vivo* and *in vitro* using lethality tests in guinea pigs, cytotoxic activity assays on HeLa cells and Vero cells and an ADP-ribosyltransferase enzymatic assay [16].

CRM_197_ is classically purified from culture supernatant of the *Corynebacterium diphtheriae* C7 (β_197_) tox negative (Tox−) strain [4]. The production of CRM_197_ from its C7 strain often suffers from low yield and also requires sophisticated laboratory conditions to cultivate the *C. diphtheriae* C7 strain. More recently, CRM_197_ has been produced in heterologous recombinant systems such as *Escherichia coli* and *Pseudomonas fluorescens* [17–19] with higher yield. The characterization of CRM_197_ protein produced from *C. diphtheriae* C7 strain has been published [15,16]. However, limited data are available for CRM_197_ produced from *E. coli* based recombinant source. Also, there is lack of information whether *E. coli* based recombinant CRM_197_ is structurally and immunologically equivalent to its incipient counterpart (C7 CRM_197_). In the present study, for the first time we have performed structural and immunological characterization of *E. coli* derived recombinant CRM_197_ protein. The data are particularly important because the increasing global demand of polysaccharide conjugate vaccines against encapsulated bacterial pathogens highlight the pressing need for high yield processes delivering well-characterized carrier protein that meets regulatory and safety requirements. The objective of present study was to: (i) express the rCRM_197_ protein from *E. coli* and its physicochemical characterization, (ii) determination of X-ray crystal structure of recombinant CRM_197_ and demonstration of its structural equivalence with C7 CRM_197_, (iii) development of polysaccharide conjugate vaccine using rCRM_197_ and demonstration of immunological functionality of rCRM_197_ as carrier protein.

## Materials and methods

### Cloning, expression and purification of CRM_197_

The codon optimized sequence corresponding to *CRM_197_* gene was synthesized and cloned into pTWIN 1 vector (New England Biolabs, U.K.) using BamH1 and Sap1 restriction sites [18]. The pTWIN1_CRM_197_ plasmid was transformed into chemically competent BL21-DE3 *E. coli* expression host. Transformants were screened on LB agar plates supplemented with ampicillin (100 µg/ml). Five to ten randomly selected colonies were purified by repeated subculturing on LB + ampicillin (100 µg/ml) plate. Finally, a few colonies were selected and a research cell bank was generated. For the expression analysis of rCRM_197_ protein, clones were grown in 100-ml LB + Ampicillin (100 µg/ml) till mid log phase (OD_600_ ~1). At this stage, culture was induced with 1 mM IPTG and was further grown to reach late log phase (OD_600_ ~4–5). One millilitre culture from each transformant was centrifuged and pellet was resuspended into 100-µl SDS loading buffer and heated at 80°C for 10 min. Twenty microlitres of lysate was loaded on 8–12% Tri-Glycine gel and SDS/PAGE was run using 80 V constant current for ~2 h. Gel was stained with Coomassie Blue (make: Amresco, Cat No: 0472-25 G). The expression of rCRM_197_ was confirmed by comparing a band appearing at ~58 kDa on the gel. Further, Western blot was performed using anti-CRM_197_ antibody (Make: Santa Cruz Biotechnology, Cat No: SC2054). One of the clones expressing rCRM_197_ protein has been used for fermentation and large-scale production of protein.

Fermentation inoculum was generated by inoculating one vial of research cell bank into seed flask containing 200-ml LB + ampicillin (100 µg/ml). Seed flask (containing growth media and inoculum) was grown to achieve OD_600_ ~4 and inoculated into 20-l fermentation medium. Fermentation was performed in chemically defined medium. After fermentation was complete, cell mass was separated by centrifugation. The cell pellet was lysed by Micro fluidizer high pressure homogenization. Homogenized cell mass was centrifuged and pellet containing inclusion body protein was retained and used for rCRM_197_ purification. The inclusion body was solubilized in buffer containing 6 M urea at 30°C and rCRM_197_ was purified by refolding followed by ion exchange chromatography [18]. The solubilized inclusion body protein was refolded and purified by ion exchange chromatography method described by Stefan et al. [19].

C7 CRM_197_ used as a reference in the studies was procured as a lyophilized powder from Sigma–Aldrich (Cat# D2189).

### Endonuclease assay for rCRM_197_

The assay was performed as described by Stefan et al. (2011) [19] with minor modifications. In brief, 2 µg rCRM_197_ was mixed with 1 µg pUC57 plasmid DNA using endonuclease reaction buffer (10 mM Tris/HCl pH 7.6, 2.5 mM CaCl_2_, 2.5 mM MgCl_2_). Content was mixed by brief centrifugation (3000 ***g***, 30 s) and incubated for different time points (0, 0.5, 1, 3, 5 and 18 h) at 25°C. The 0-h time point sample was used as a control. At each time point, reaction was terminated by the addition of 5 mM EDTA (final concentration) to each tube and frozen at −20°C. Thawed samples were mixed with DNA loading buffer and run on 1% agarose gel electrophoresis in 1× TAE buffer using 80 V constant voltage. DNA staining dye SYBR Safe gel stain (Invitrogen, Cat# S33102) was added to the agarose gel at the time of pouring. After the electrophoresis run was completed, the gel was analysed under UV transillumination (Bio-Rad ChemiDoc Gel imaging System).

### SDS/PAGE and Western immunoblot analysis

Reducing and nonreducing SDS/PAGE and Western blot analyses were performed with purified rCRM_197_ as per the standard protocol [20]. Four micrograms of purified rCRM_197_ along with the reference CRM_197_ was loaded on to 4–12% Tris-Glycine gels under reducing and nonreducing conditions. Gels were run at 80 V constant voltage until blue dye reached the bottom of the gel. SDS/PAGE molecular weight markers (New England Biolabs, Cat# P7712S) were used for molecular weight calibration. After Coomassie Brilliant Blue staining, the image was captured using transilluminator (Bio-Rad ChemiDoc system). For Western immunoblotting, 2 μg protein was separated by SDS/PAGE and transferred to nitrocellulose membrane (0.45 µm, Bio-Rad, Cat# 1620115). The blot was probed with α-CRM_197_ rabbit polyclonal primary antibody (Abcam, Cat# ab151222) by incubating the membrane for 1 h. After the primary incubation, the membrane was further incubated in HRP-conjugated secondary antibody (rabbit) (make: Santa Cruz Biotechnology, Cat No: SC2054). Bands were visualized using 3,3′-diaminobenzidine (DAB) substrate and the image captured using Bio-Rad ChemiDoc gel imaging system.

### Intact MS

The intact mass of rCRM_197_ was determined by LC-MS (ABSCIEX Triple TOF 5600+) using the published protocol [21,22]. Protein samples were buffer exchanged by centrifugal ultrafiltration (Amicon Ultra-0.5 ml, 10 K MWCO, 14000 ***g***, 15 min, 4°C) and analysed by LC-qToF using the manufacturer’s protocol.

### Amino acid composition analysis

rCRM_197_ was subjected to high sensitivity amino acid analysis (AAA). The analysis was performed on samples desalted by ultrafiltration (Vivaspin 6 spin columns with a 10-kDa polyethersulfone filter: Sartorius, Cat# VS0601). Samples were resuspended in 20% acetonitrile (ACN) containing 0.1% trifluoroacetic acid (TFA) and subjected to gas phase hydrolysis (6 N HCl at 110°C). α-amino butyric acid (AABA) was run as an internal standard prior to sample analysis. Cysteine analysis was performed after performic acid oxidation followed by hydrolysis as above. Hydrolysates were analysed using a Waters Acquity UPLC system with AccQTag Ultra chemistry. Samples were analysed in duplicate and the mean reported [23,24].

### Western immunoblotting with structural monoclonal antibodies against CRM_197_/DT

rCRM_197_ samples along with reference were run in SDS/PAGE. The gel was transferred to nitrocellulose membrane and Western immunoblotting was done using five different monoclonal antibodies (mAbs) against DT/CRM_197._ These mAbs were 7F2 (Abcam, Cat. No: ab8310), 8A4 (Abcam, Cat. No: ab8308), 3B6 (Abcam, Cat No: ab8307), 1H2 (Abcam, Cat. No: ab8306) and HYB 123-09 (Statens Serum Institute, Cat. No: 66045). The SDS/PAGE and Western blot methods were as described above.

### Determination of crystal structure of rCRM_197_ by X-ray crystallography

Center for the Structural Genomics of Infectious Diseases (CSGID) standard protocols were used to determine the structure of CRM_197_ [25–27].

Sitting drop crystallization plates were set up at room temperature and crystals of rCRM_197_ were obtained by mixing 22.9 mg/ml of protein in 0.15 M sodium chloride, 0.01 M Tris/HCl buffer (pH 8.3) with the crystal screen solution of 0.2 M lithium sulfate, 0.1 M Bis-Tris buffer (pH 5.5), 25% (w/v) PEG 3350 (Qiagen, Classics II suite (G2), in a 1:1 ratio. Harvested crystals were transferred to the reservoir solution before being flash-frozen in liquid nitrogen. Diffraction data were collected at 100 K at the Life Sciences Collaborative Access Team at the Advance Photon Source, Argonne, Illinois (APS BEAMLINE 21-ID-F). Data were processed using HKL-3000 for indexing, integration and scaling. The crystal structure of recombinant CRM_197_ (Protein Data Bank (PDB) ID: 5I82) was determined by molecular replacement using PDB entry 4AE0 as a search model. The structure was refined with Refmac 5 [28]. Models were displayed in Coot and manually corrected based on electron density maps [29]. All structure figures were prepared using PyMOL Molecular Graphics System, version 1.3 (Schrödinger, LLC). The structure was submitted to the PDB (PDB_ ID: 5I82).

### Generation of typhoid conjugate vaccine (Vi-rCRM_197_) using rCRM_197_ and immunogenicity assessment

rCRM_197_ was conjugated with Vi polysaccharide antigen to generate Vi-rCRM_197_ conjugate vaccine (typhoid conjugate vaccine). The Vi polysaccharide was produced by fermentation of *Citrobacter freundii* as per method described by Rondini et al. [30]. The *C. freundii* stain was provided by Dr Laura B. Martin, GSK Vaccine Institute of Global Health, Siena, Italy. The Vi-rCRM_197_ conjugate was prepared according to the method reported by Kossaczka et al. [31] and as detailed by Micoli et al. [8]. Briefly, rCRM_197_ was derivatized with adipic acid dihydrazine (ADH). Then, Vi (purified from *C. freundii*) was activated with N-hydroxy succinamide and 1-ethyl-3(3-dimethylaminopropyl) carbodiimide hydrochloride and linked to the derivatized rCRM_197_ protein generating Vi-rCRM_197_ conjugate vaccine. The ADH derivatized rCRM_197_ (rCRM_197_-ADH) was mixed with activated Vi polysaccharides in 1:1 ratio and incubated at room temperature for 5–6 h. The resulting conjugate was purified by HIC (hydrophobic interaction chromatography) using phenyl sepharose (GE) resin.

The immunogenicity of Vi-rCRM_197_ was performed as per procedures described by Rondini et al. [30]. Four to six weeks old female Balb/C mice were used in the experiment. A group of 30 mice were subcutaneously immunized with three doses of Vi-rCRM_197_, unconjugated Vi polysaccharide and PBS (placebo control). A 100μl dose of Vi-rCRM_197_ conjugate vaccine containing 2.5 µg Vi, unconjugated Vi polysaccharide (2.5 µg) and PBS (control) were administrated in mice on day 1 followed by booster dose at days 14 and 21. Fourteen days after first immunization, ten randomly selected mice from each group were terminally bled to collect the post-dose 1 sera. Remaining animals (20 each) were given the second dose of vaccine. Seven days after second immunization (day 21), ten mice from each group were terminally bled and post-dose 2 sera was collected. Finally, remaining ten mice were given the third dose of vaccine. The final sera (post-dose 3) was collected at day 28 by bleeding of remaining mice from each group. Mice sera was subjected to ELISA analysis and anti-Vi IgG response was measured as per the procedure described by Micoli et al. [8].

## Results

### Determination of molecular weight and identity of *E. coli* derived rCRM_197_

**Figure 1:**
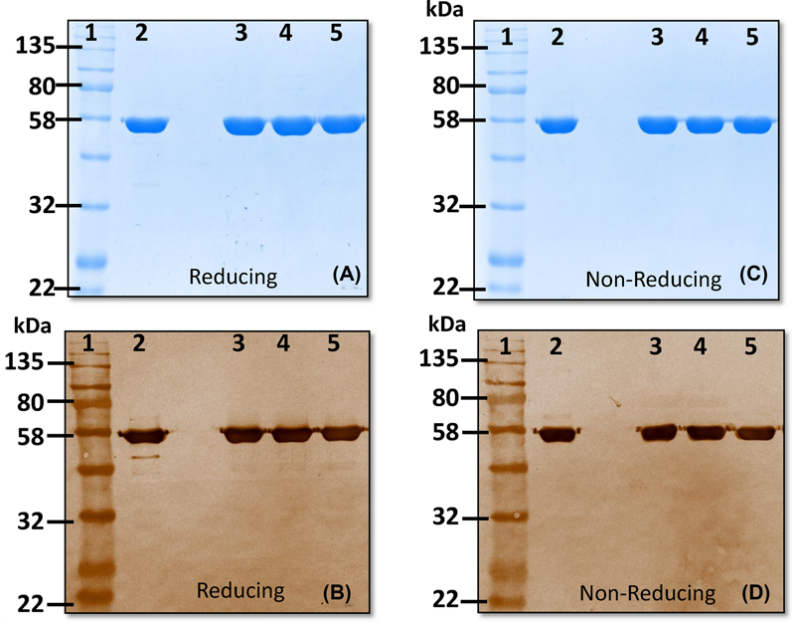
Determination of molecular weight, identity and purity of CRM_197_ by SDS/AGE and Western immunoblot respectively (**A,B**) Reducing SDS/PAGE and Western blot analysis of three different lots of rCRM_197_, and reference CRM_197_. (**C,D**) Nonreducing SDS/PAGE and Western blot analysis of three different lots of rCRM_197_ and reference CRM_197_ (Lane 1: protein molecular weight marker, Lane 2: reference CRM_197_, Lane 3–5: rCRM_197_ Lot 1–3). Purified recombinant CRM_197_ protein appeared as ~58 kDa molecular weight protein on SDS/PAGE with ≥95% purity. This molecular weight matches the expected size of the protein and with reference standard used. Anti-CRM_197_ antibodies recognized rCRM_197_ in Western blot, thereby confirming the protein identity as CRM_197_.

rCRM_197_ appears as a ~58 kDa band in SDS/PAGE and Western blot analyses performed under both nonreducing and reducing conditions. This is consistent with the reported molecular weight of CRM_197_ of 58.4 kDa [16]. Gels run under nonreducing conditions help to evaluate possible covalent and noncovalent aggregation in the samples and to detect impurities. rCRM_197_ contains two disulfide bonds, one linking chains A and B and a second within the B chain. Gels run in reducing conditions allow detection of the proteolytically nicked form (if any) of the protein. β-mercaptoethanol (2-βME) was used to reduce disulfide bridges during sample preparation. Under these reducing conditions, rCRM_197_ (test sample) showed no visible evidence of nicked and/or degraded forms in SDS/PAGE (Figure 1A,B). Figure 1C,D summarizes the Western blot analysis of rCRM_197_ and reference CRM_197_ run in reducing and nonreducing conditions. The Western blot was probed with α-CRM_197_ rabbit polyclonal antibody (make: Abcam; Cat No: ab151222).

Three independent manufacturing lots of rCRM_197_ were evaluated in the present study and exhibited similar profiles under both nonreducing and reducing conditions, also supporting consistency of manufacturing for rCRM_197_.

### Intact mass determination by MS

**Figure 2:**
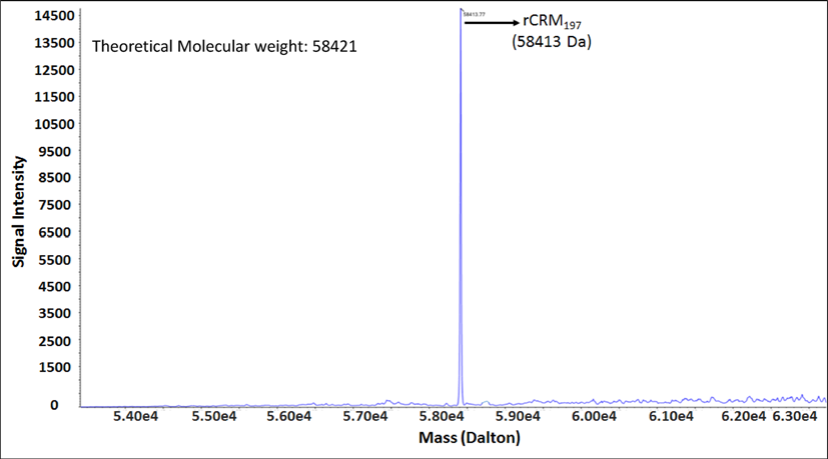
Determination of intact mass of rCRM_197_ using MS (ESI-qTof) The major abundant peak of 58.4 kDa has been observed which is similar to the expected/theoretical molecular weight of CRM_197_ protein.

MS is a powerful analytical tool as it measures an intrinsic property of a molecule, its mass, with very high sensitivity and therefore it is used in a wide range of applications [22]. The intact mass of rCRM_197_ was obtained by deconvolution of mass/charge (m/z) ratio. Data obtained by TOF-MS showed most abundant mass of 58413 Da compared with a theoretical calculated mass of 58421 Da based on amino acid sequence (Figure 2). This result confirms the molecular weight of the purified rCRM_197_ protein i.e. ~58.4 kDa. Similar data have also been reported both for a recombinant CRM_197_ produced in *Pseudomonas fluorescens* and C7 CRM_197_ [21,32].

### Endonuclease activity

**Figure 3:**
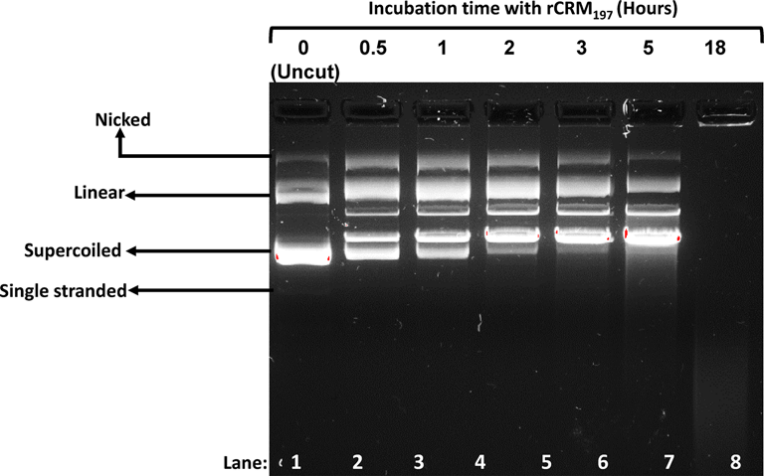
Demonstration of functional activity in rCRM_197_ samples purified from inclusion body pUC57 plasmid was incubated with rCRM_197_ for different time points and the mix was run on 1% agarose gel. Native CRM_197_ possesses a mild endonuclease activity and this assay is used to prove the restoration of biological activity and conformation integrity of the protein purified from insoluble inclusion body. The purified rCRM_197_ exhibits the endonuclease activity as evident by nicking of supercoiled plasmid DNA incubated with the protein (Lanes 2–7).

Mild endonuclease activity is an inherent property of CRM_197_ and its parent molecule DT [4] and is considered as an indicator of the correctly folded bioactive form of the protein. Incubation of CRM_197_ with supercoiled plasmid DNA in the presence of divalent cations (e.g. Ca^++^ and Mg^++^) results in DNA cleavage [19,33]. Incubation of pUC57 plasmid DNA with rCRM_197_ in a 2:1 ratio for different times and analysis of the DNA by agarose gel electrophoresis confirmed the positive nuclease activity in the protein. During incubation, rCRM_197_ was able to linearize the supercoiled DNA. No endonuclease activity was seen in the 0-h time point sample but it increased with the incubation time (Figure 3). This endonuclease assay can be used as an in-process control assay for CRM_197_ protein produced from insoluble inclusion bodies. Similarly, earlier studies in which endonuclease activity was used to establish the purity and correctness of refolding of rCRM_197_ produced in *E. coli* is also reported [19]. Previous reports also showed that both DT and CRM_197_ should have fragment A-associated nuclease activity [33].

### Determination of amino acid composition

**Figure 4:**
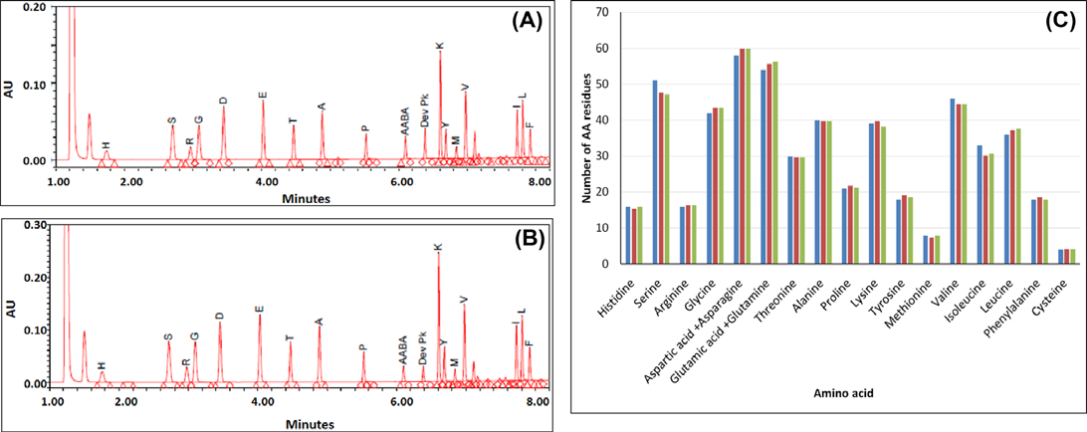
High-sensitivity AAA profile of rCRM_197_ and CRM_197_ Both the proteins showed similar elution profile during AAA chromatogram of high-sensitivity AAA of CRM_197_. (**A**) AAA chromatogram of BioE rCRM_197_ and (**B**) AAA chromatogram of reference CRM_197_. Both the proteins showed similar elution profile. The peak data were extrapolated and plotted in bar diagram (**C**). Blue bar: theoretical number of amino acid residues, Red bar: experimental amino acid residues of rCRM_197_ and Green bar: experimental amino acid residues of reference CRM_197_.

Acid hydrolysis followed by high-sensitivity AAA has been performed with rCRM_197_ and compared with reference CRM_197_. After acid hydrolysis, amino acid mixture was analysed by ultra performance liquid chromatography (UPLC) using the Waters AccQTag Ultra chemistry. All samples were analysed in duplicate and results expressed as an average. The amino acid composition of rCRM_197_ closely matches (within 1–2% variability range) with reference and with the theoretical values of CRM_197_ protein (Figure 4). AAA can be used to confirm the primary amino acid composition of protein. The overall data suggest that rCRM_197_ produced in *E. coli* has similar amino acid composition as CRM_197_ produced by *C. diphtheriae* C7 strain (reference CRM_197_) [15].

### Immunoblotting with α-CRM_197_ mAbs

**Figure 5:**
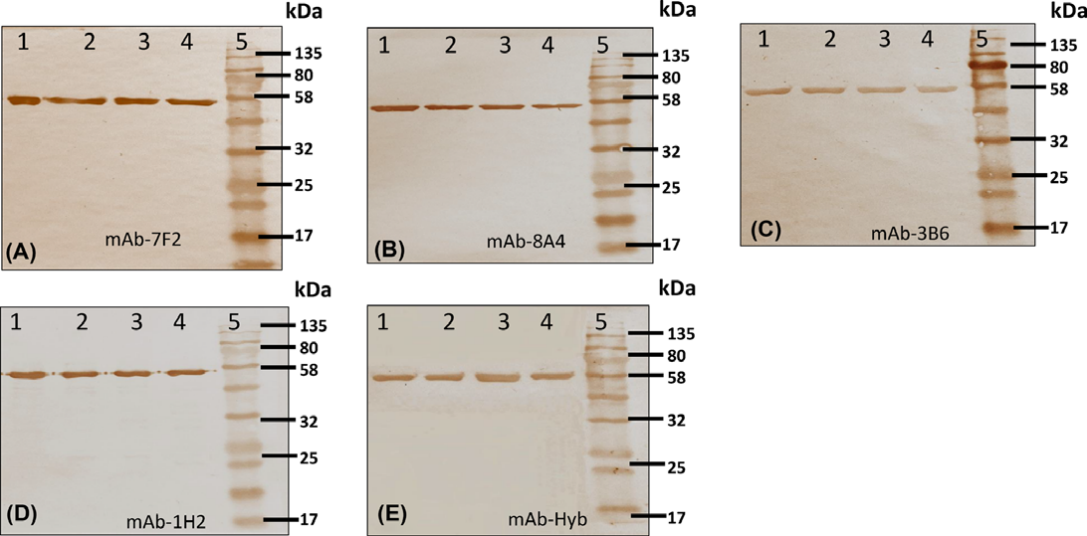
Mapping of rCRM_197_ protein by structural mAbs against CRM_197_ by Western immunoblot All mAbs recognized rCRM_197_ as single major band of ~58 kDa. This confirms the structural integrity and identity of rCRM_197_ and its equivalence with reference. (**A**,**B**) Western blot using A chain specific α-CRM_197_ mAb 7F2 and 8A4; (**C**) Western blot using B chain specific α-CRM_197_ mAb 73B6; (**D**,**E**) Western blot using whole CRM_197_ specific α-CRM_197_ mAb 1H2 and Hyb. Lane 1: Reference CRM_197_, 2–4: rCRM_197_ Lot 1–3 and Lane 5: protein molecular weight marker.

Three independent lots of rCRM_197_ were run in SDS/PAGE and were probed with five different mAbs with specificity to different epitopes on the protein in Western immunoblotting experiments. All these mAbs recognized the protein at similar level and showed a major immune-reactive band at ~58 kDa, the expected size of the protein (Figure 5). The reference CRM_197_ was recognized at similar level as rCRM_197_. However, a slight difference in the recognition pattern was observed with respect to different mAbs, which suggests differential affinity of different mAbs for recognition of CRM_197_. mAb mapping of CRM_197_ has been found useful to assess potential nicking of A and B chains that could be seen in Western blot. A similar study was performed with different mAbs raised against CRM_197_ for their ability to bind DT and six functional mutants CRM_197_, CRM_176_, CRM_228_, CRM_1001_, CRM_45_ and CRM_30_ by immunoblotting [35]. The results showed a difference in the recognition pattern of mAbs specific to different part of the protein. Our data are of particular importance because protein is purified from insoluble inclusion body after refolding. There is potential chance of misfolding and or improper folding that might impact the conformation of the protein. Hence, the data also confirm that rCRM_197_ has attained the correct conformational structure after purification from insoluble inclusion bodies [35].

### Determination of crystal structure of *E. coli* rCRM_197_ by X-ray crystallography

The crystal structure of *E. coli* derived recombinant CRM_197_ was determined at 2.3 Å resolution and the structure was submitted to the PDB with the PDB ID: 5I82. Previous studies have elucidated the structural features of DT in different conditions, such as in the *apo* conformation, at acidic pH, in complex with NAD, with dinucleotide inhibitor adenylyl 3′–5′ uridine 3′-monophosphate and with an extracellular fragment of its receptor [36–39]. More recently, the structures of C7 CRM_197_ in the *apo* form and in complex with the NAD hydrolysis product nicotinamide have been determined [16]. This showed that the single amino acid substitution of G52E that is the difference between CRM_197_ and DT, results in an intrinsic flexibility of the active-site loop [16]. All these studies were performed on DT and CRM_197_ produced by *C. diphtheriae*. However, there is very limited information available on CRM_197_ protein produced from the recombinant *E. coli*. To verify if the rCRM_197_ produced from insoluble inclusion body protein have the overall similar 3D structure of the protein, we determined the X-ray crystal structure of rCRM_197_. Furthermore, the crystal structure of rCRM_197_ confirms the presence of structural determinants that ablated the toxicity of DT and resulted in CRM_197_ (G52E mutation).

**Figure 6:**
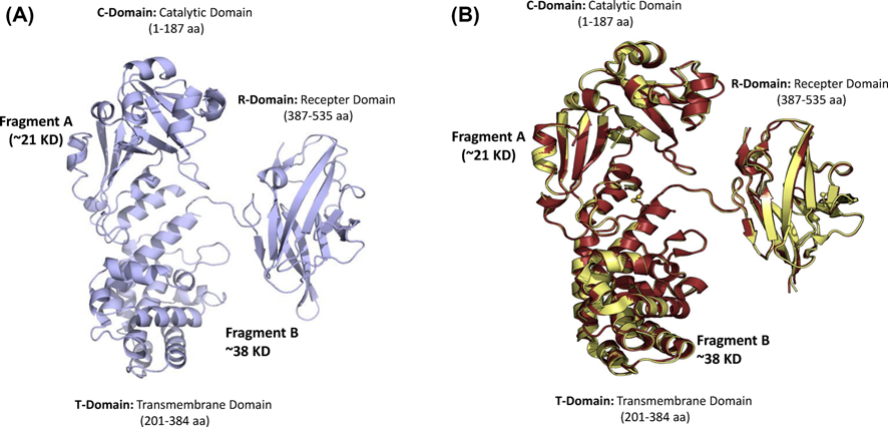
Crystal structure of monomeric form of *E. coli* derived rCRM197 protein (PDB ID: 5I82) (**A**) Superimposition of the monomer of rCRM_197_ and C7 CRM_197_. (**B**) Chain A (cartoon colored as ruby) and chain B (transparent, dark grey ribbon) of rCRM_197_ are superimposed on the monomer of wild-type CRM_197_ (PDB code: 4AE0, cartoon colored as yellow) and its symmetry-related molecule (transparent, yellow ribbon). (C) Catalytic domains consisting of residues 1–187, (T) Transmembrane domain consisting of residues 201–384, (R) Receptor domain consisting of residues 387–535. The dimers show the swapping of the T domains in the open conformation.

The structure was determined at 2.3 Å resolution by molecular replacement using a search model, the coordinates of the *apo* form of C7 CRM_197_ (PDB entry 4EA0). Data collection, refinement and the final model validation statistics for the structure of rCRM197 (PDB ID: 5I82) have been given in Table 1. The asymmetric unit contains four molecules of rCRM_197_ (chains A, B, C and D). The four chains interact as dimers (chains A–B and chains C–D) by domain swapping, with each monomer in ‘open’ conformation, a typical packing that has already been described for both DT and CRM_197_ (Figure 6) [16]. The overall fold of each chain is similar to C7 CRM_197_ (PDB: 4AE0). In fact, by superimposing the single chains or the two homodimers (chains A–B and C–D), the RMSD results were less than 0.17 Å (A and B, 428 out of 507 Cα, RMSD of 0.17 Å; A and C, 424 out of 508 Cα, RMSD of 0.17 Å; A and D, 426 out of 504 Cα, RMSD of 0.10 Å; AB and CD, 839 out of 1011 Cα, RMSD of 0.17 Å) [40], demonstrating that these are very similar.

**Table 1:** Refinement statistics of CRM_197_ (PDB ID: 5182)

***Data collection statistics***	
Space group	P1
Unit cell: *a, b, c* (Å) *α, β, γ* (°°)	69.21, 69.17, 127.88, 90.09, 90.01, 82.02
Wavelength (Å)	0.97872
Resolution (Å)	30.00–2.35 (2.39–2.35)*
Number of observed reflections	98137 (4888)
R*_merge_* (%)	5.1 (42.2)
Completeness (%)	98.4 (97.7)
*I/σ*(*I*)	18.2 (2.3)
Redundancy	2.6 (2.6)
***Refinement and validation***	
Resolution (Å)	29.08–2.35 (2.41–2.35)
*R*_work_/*R*_free_ (%)	20.7 (27.2)/25.0 (32.9)
Number of atoms	
Protein	15730
Ligand/Ion	21
Water	351
B-factors	
Protein/ligand/water	66.6/94.2/54.3
RMSD bond lengths (Å)	0.007
RMSD bond angles (°)	1.174
***PDB ID***	5I82

The dimers in the crystal structure of rCRM_197_ and C7-CRM_197_, are equivalent. By superimposing the A and B chains of rCRM_197_ on to C7-CRM_197_ and its symmetry-related molecule that forms the dimer, 924 out of 998 Cα superimposed with RMSD of 0.38 Å (Figure 6). The overall fold of the monomer rCRM_197_ is equivalent to C7-CRM_197_, with an overall RMSD of 0.34 Å for 451 out of 498 superimposed Cα atoms (Figure 6). Since overall structure is preserved between C7 CRM_197_ and rCRM_197_, it is unlikely that there will be any significant differences between these two molecules in terms of T epitopes that can potentially impact the efficacy of rCMR_197_.

**Figure 7:**
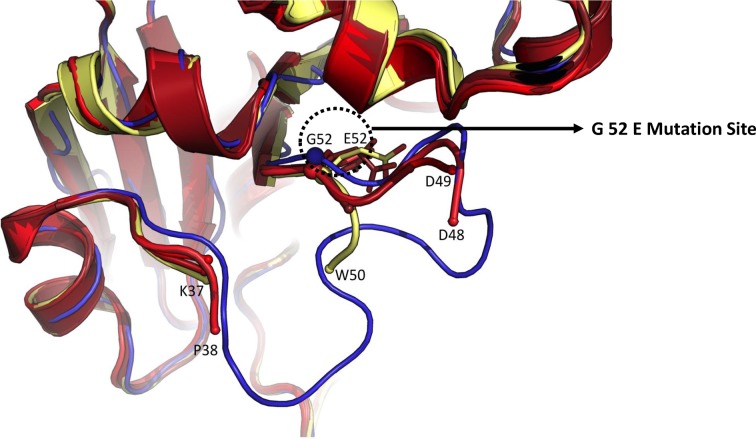
The glycine to glutamate mutation at amino acid position 52 (G52E) in rCRM_197_. Zoom in the active site loop CL2 where G52E mutation has taken place. The mutation creates a structural distortion in active site loop of DT thereby eliminating its toxicity (consequently CRM_197_ is formed). The image depicts the structural superimposition of the DT (PDB code: 1SGK; thin blue ribbon), C7-CRM_197_ (PDB code: 4AE0, yellow carton) and rCRM_197_ (PDB code: 5I82: chain A as ruby cartoon, chain B as red cartoon, chain C as light red cartoon and chain D as firebrick color cartoon). The data demonstrate that rCRM_197_ possesses G52E point mutation that converted DT into CRM_197_. The same point mutation was also evident in C7-CRM_197_.

Furthermore, previous studies showed that the substitution G52E induces the dislocation of the active-site loop CL2 in C7-CRM_197_ with respect to DT [16]. The intrinsic flexibility of CL2 results in a localized low quality electron density map in the C7-CRM_197_ structure [38,39]. In our crystal structure of rCRM_197_, we have also observed a low electron density in the active loop CL2, consistent with the previous results (Figure 7). This confirms the structural basis of lack of toxicity in rCRM_197._

**Figure 8:**
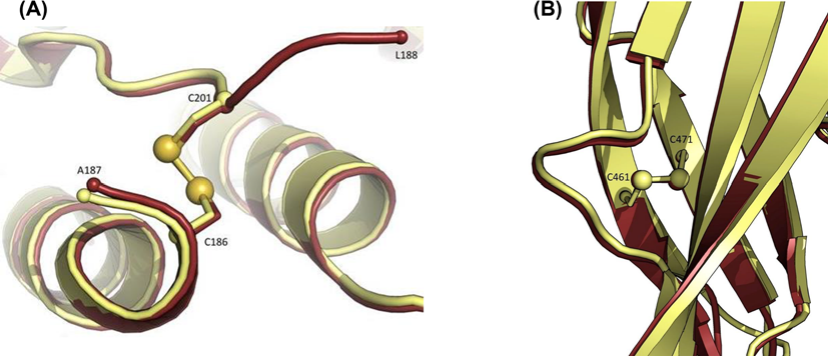
The presence of correct dilsuphide bonds in *E. coli* derived rCRM_197_. Zoom in the disulfide bond C186-C201 (**A**) and C461-C471 (**B**) in rCRM_197_ (PDB ID: 5I82). Two disulfide bonds has been identified in at the expected position of the resolved structure of rCRM_197_.

Another important biochemical and structural features of DT, as well as C7 CRM_197_, is the presence of the disulfide bond C186-C201 that maintains the A and B fragments bound together in the nicked form after the proteolytic cleavage of the chain. The structure revealed that the disulfide bond is conserved in rCRM_197_ (Figure 8).

To our knowledge, this is the first reported crystal structure of *E. coli* derived rCRM_197_ and first described comparison with C7-CRM_197_. In conclusion, rCRM_197_ conserves the overall folding, making it structurally equivalent to C7-CRM_197_, which is used in many licenced conjugate vaccine such as meningitis, pneumococcal conjugate vaccine etc. [10–14].

### Immunogenicity of Vi-rCRM_197_ conjugate vaccine in mice

**Figure 9:**
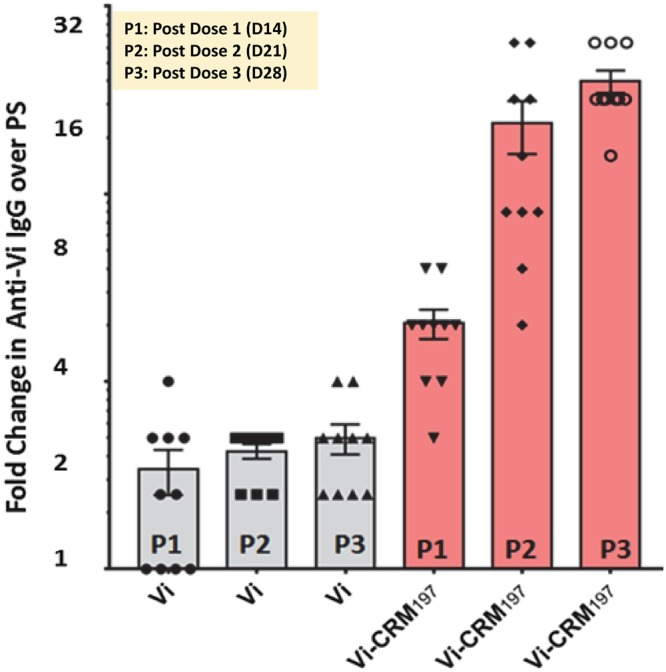
Immunogenicity of typhoid conjugate vaccine (Vi-rCRM_197_) generated by conjugating Vi polysaccharide with rCRM_197_ Balb/C mice immunized with three dose of Vi-rCRM_197_ conjugate vaccine and Anti Vi-IgG was measured by ELISA. The titre of Vi specific antibody was compared with Vi PS (unconjugated Vi polysaccharide) group. There was significant increase in the Vi antibody was observed in Vi-CRM_197_ immunized mice and a booster response was also observed after second injection.

Immunogenicity of typhoid conjugate vaccine (Vi-rCRM_197_) generated using *E. coli* derived rCRM_197_ as carrier protein was evaluated in Balb/c mice. Mice were immunized with three doses of conjugate vaccine Vi-rCRM_197_ and anti-Vi IgG immune response was measured by ELISA. Sera from mice immunizied with PBS was used to generate the baseline. Vi-rCRM_197_ immunized mice were able to elicit strong Anti-Vi IgG antibody response. Mice immunized with unconjugated Vi polysaccharide (Control) showed only basal level of Anti-Vi IgG. The Anti-Vi antibodies response also found to be bootable as seen in a steep increase in IgG titre after second dose of vaccine (Booster) (Figure 9). The Anti-Vi IgG response was in mice immunized with Vi-rCRM_197_ conjugate vaccine was found to be ~16-fold higher than teh baseline response (PBS) after booster dose wheseas un-conjugated Vi showed only ˜ 2 fold increase in the anti-Vi IgG over baseline. Noteworthy, the response of anti-Vi IgG in mice immunized with conjugate vaccine was 10-12 fold higher than the un-conjugated Vi.

As per the recommendation of World Health Organization-Technical Report Series (WHO-TRS) on typhoid conjugate vaccine, the conjugate vaccine should induce an immune response that is at least four-fold higher than the response induced by un-conjugated Vi, and a booster response should occur after the second dose in animals (WHO-TRS, 2013) [41]. The conjugate vaccine generated by using rCRM_197_ as carrier protein comfortably meets the WHO immunogenicity criteria for typhoid conjugate vaccine, as it induces more than four-fold Anti-Vi IgG and also exhibits a booster response in mice. The data confirm the immunological functionality of rCRM_197_ as carrier protein and suggests that it is able to convert T-independent response of polysaccharides in mice into T-dependent one and therefore booster antibody response is resulted. These data corroborate that rCRM_197_ conjugation with Vi polysaccharide in mice can be resulted in immunogenic conjugates and therefore strengthening the suitability of rCRM_197_ as a carrier protein for the development of conjugate vaccines.

## Conclusion

The present study provides the detailed structural and immunological characteristics of CRM_197_ carrier protein produced by heterologous expression in *E. coli.* In addition to reveal the scientific insights of rCRM_197_, the data presented in this work are also important in the context that regulatory guidelines on conjugate vaccines that emphasizes the need for in-depth characterization of carrier protein used in the conjugate vaccines development. Successful and broadly marketed carbohydrate conjugate vaccines are based on just a few FDA-approved carrier proteins. First-generation of carrier proteins such as DT and tetanus toxin require detoxification with formaldehyde, potentially eliminating part of the lysine residues needed for glycan attachment, thereby potentially compromising the conjugation efficacy. CRM_197_ is one of the most widely used and highly effective carrier protein for conjuate vaccine. Licenced conjugate vaccines such as HibTITER (*Haemophilus influenzae* type b associated diseases), Prevnar (pneumococcal diseases), and Menveo (meningococcal diseases) are containing CRM_197_ as a carrier protein. Majority of currently marketed glycoconjugate vaccines contain CRM_197_ as a carrier protein and access to high quality material will support the development and production of new conjugate vaccines. The data presented herein demonstrate for the first time structural and immunological characterization of rCRM_197_ produced in *E. coli*.

In a recent study, the recombinant CRM_197_ has also been expressed in *E. coli* using pET28a expression vector [42]. Unlike our study, CRM_197_ was expressed in Histidine-tagged form (*His*-CRM_197_) which requires a histidine removal step by enterokinase treatment. This process often poses the limitation during the scale-up of production process and inconsistencies with respect to batch to batch purity/impurity profiles of protein. The protein was purified at small scale by immobilized metal affinity chromatography (IMAC) with reasonable purity [42]. However, the in-depth analytical characterization of protein was also lacking in this study. In the present study, rCRM_197_ expression was done in pTWIN expression vector, which releases the expressed protein from fusion tag by self-splicing with high fidelity. CRM_197_ protein was purified at high scale (~20 l Fermentation Scale) using ion exchange chromatography and highly pure form of protein (>95%) was recovered as the final product.

Recently, we have studied the analytical comparability of five CRM_197_ proteins produced in three different expression systems (*E. coli, P. fluorescens* and *C. diphtheriae* C7). A comprehensive physicochemical analysis of the CRM_197_ molecules demonstrate that recombinant CRM_197_s expressed in *E. coli* are overall highly similar to those expressed in the traditional system (*C. diphtheriae* C7) in terms of primary sequence/post-translational modifications, higher order structural integrity, apparent solubility, physical stability profile and *in vitro* antigenicity [43]. However, the detailed structural analysis and its *in vivo* antigenicity/immunological characterization was lacking. As a part of further in-depth characterization, we have determined the 3D crystal structure of *E. coli* derived rCRM_197_. The rCRM_197_ has shown the equivalence with C7 CRM_197_ in terms of overall folding pattern, domain architecture and the presence of correct disulfide bonds. The absence of toxicity in CRM_197_ relies on a point mutation at amino acid position 52 that replaces Glycine with Glutamate (G52E) and renders protein nontoxicity. In the present study, the genetic basis of nontoxicity of *E. coli* based rCRM_197_ was also confirmed by X-ray crystallography. These data are important for scientific communities as well as industries working on conjugate vaccine development using CRM_197_ as carrier protein. The *in vivo* functionality of *E. coli* expressed recombinant CRM_197_ carrier protein has been demonstrated by conjugating it with Vi polysaccharide antigen of *Salmonella* Typhi thereby generating Vi-rCRM_197_ conjugate vaccine. The data confirm that the use of rCRM_197_ as a carrier protein in conjugate vaccine will be able to elicit the primary and a booster antibody response in animals. The Anti-Vi IgG elicited by Vi-rCRM_197_ conjugate vaccine was more than four-fold higher than the response generated by unconjugated Vi which is one of the benchmark for a protective typhoid conjugate vaccine [41]. The comparative immunogenicity of Vi polysaccharide conjugated to other carrier protein like tetanus toxoid (TT), diphtheria toxoid (DT) and C7 CRM_197_ is already published [44]. The immunogenicity of Vi-rCRM_197_ showed the comparable result in terms of overall fold change in Anti-Vi IgG over Vi and booster impact. The overall data presented in our study will potentially catalyse the efforts for the production of CRM_197_ protein in the recombinant system and its use as carrier protein in the conjugate vaccine development.

## Abbreviations

AAA: amino acid analysis
ADH: adipic acid dihydrazine
ADP: adenosine diphosphate
CRM_197_: cross reacting material 197
CSGID: Center for the Structural Genomics of Infectious Diseases
DT: diphtheria toxin
FDA: Food and Drug Administration
HB-EGF: heparin binding epidermal growth factor
HRP: horseradish peroxidase
LC-qTOF: liquid chromatograpy-quadrupole time-of-flight
mAb: monoclonal antibody
PDB: Protein Data Bank
TAE: Tris-acetate-EDTA
WHO-TRS: World Health Organization-Technical Report Series
